# In Vitro Acute Exposure to DEHP Affects Oocyte Meiotic Maturation, Energy and Oxidative Stress Parameters in a Large Animal Model

**DOI:** 10.1371/journal.pone.0027452

**Published:** 2011-11-04

**Authors:** Barbara Ambruosi, Manuel Filioli Uranio, Anna Maria Sardanelli, Paola Pocar, Nicola Antonio Martino, Maria Stefania Paternoster, Francesca Amati, Maria Elena Dell'Aquila

**Affiliations:** 1 Department of Animal Production, University of Bari Aldo Moro, Valenzano, Bari, Italy; 2 Department of Medical Biochemistry, Biology and Physics, University of Bari Aldo Moro, Bari, Italy; 3 Dipartimento di Patologia Animale, Igiene e Sanità Pubblica Veterinaria, University of Milan, Milan, Italy; University Paris Diderot-Paris 7, France

## Abstract

Phthalates are ubiquitous environmental contaminants because of their use in plastics and other common consumer products. Di-(2-ethylhexyl) phthalate (DEHP) is the most abundant phthalate and it impairs fertility by acting as an endocrine disruptor. The aim of the present study was to analyze the effects of *in vitro* acute exposure to DEHP on oocyte maturation, energy and oxidative status in the horse, a large animal model. Cumulus cell (CC) apoptosis and oxidative status were also investigated. Cumulus-oocyte complexes from the ovaries of slaughtered mares were cultured *in vitro* in presence of 0.12, 12 and 1200 µM DEHP. After *in vitro* maturation (IVM), CCs were removed and evaluated for apoptosis (cytological assessment and TUNEL) and intracellular reactive oxygen species (ROS) levels. Oocytes were evaluated for nuclear chromatin configuration. Matured (Metaphase II stage; MII) oocytes were further evaluated for cytoplasmic energy and oxidative parameters. DEHP significantly inhibited oocyte maturation when added at low doses (0.12 µM; P<0.05). This effect was related to increased CC apoptosis (P<0.001) and reduced ROS levels (P<0.0001). At higher doses (12 and 1200 µM), DEHP induced apoptosis (P<0.0001) and ROS increase (P<0.0001) in CCs without affecting oocyte maturation. In DEHP-exposed MII oocytes, mitochondrial distribution patterns, apparent energy status (MitoTracker fluorescence intensity), intracellular ROS localization and levels, mt/ROS colocalization and total SOD activity did not vary, whereas increased ATP content (P<0.05), possibly of glycolytic origin, was found. Co-treatment with N-Acetyl-Cysteine reversed apoptosis and efficiently scavenged excessive ROS in DEHP-treated CCs without enhancing oocyte maturation. In conclusion, acute in vitro exposure to DEHP inhibits equine oocyte maturation without altering ooplasmic energy and oxidative stress parameters in matured oocytes which retain the potential to be fertilized and develop into embryos even though further studies are necessary to confirm this possibility.

## Introduction

Phtalates are a family of industrial compounds used as plasticizers in the manufactures of many products such as infant toys, building and food packaging materials, and biomedical devices [1]. These plasticizers are not covalently bound to the polymer and leach out into the environment, thus becoming ubiquitous environmental contaminants [2]. Humans are exposed to these compounds through ingestion, inhalation, and dermal exposure for their whole lifetime, since the intrauterine life [3], [4]. Among phtalates, the di-(2-ethylhexyl) phthalate (DEHP) is the most widely used [5], [6]. This agent is rapidly hydrolyzed to produce its major metabolite mono (2-ethylhexyl) phthalate (MEHP). Both DEHP and MEHP are reported as potent reproductive toxicant and they impair fertility by acting as endocrine disruptors, thus causing gonadal mophological or functional alterations in both sexes [7]. Despite experimental data provide good evidence that MEHP is highly active in mediating many of the effects of DEHP, in vitro studies have recently demonstrated that monoesters (such as MEHP) did not enter the cells as readily as did the diesters (DEHP), possibly because the charged molecules cannot pass the plasma membrane [8]. Furthermore, in vitro studies, largely conducted in cell lines or primary cell cultures, have demonstrated that DEHP is active at a cellular level, indicating either that DEHP itself has some intrinsic activity in mediating the observed effects, or that cells have some capacity for conversion of DEHP to MEHP [9].

In studies in rats, DEHP [10] has been shown to suppress granulosa cell estradiol production with consequent alteration of the gonadic-hypothalamus feedback, modifications of follicle stimulating hormone (FSH) and luteininzing hormone (LH) levels, prolonged estrous cycles, absence of ovulation and corpus luteum formation and ovarian degeneration. Biological action mechanisms of phthalates are not clearly understood besides their known ability to activate the PPAR nuclear receptors which are known to be expressed in granulosa and theca cells [11]. Until now, few studies focusing on the impact of phthalates on meiotic maturation have been reported. The first study, performed in bovine oocytes, demonstrated that the addition of MEHP, during in vitro maturation (IVM), inhibits meiotic maturation in a dose-dependent manner [1]. This result was confirmed in a subsequent study performed in mouse oocytes [12] whereas no effect was noticed by adding DEHP in IVM culture of pig oocytes [13]. Eimani et al., 2005 [14] reported inhibition of meiotic maturation in the mouse after in vivo oral DEHP administration. A very recent study in zebrafish [15] firstly reported deleterious effects of DEHP on molecular biomarkers of oocyte growth, maturation and ovulation.

It has been reported that oxidative stress (OS) may be an important mechanism underlying the toxic effects of DEHP [16]-[18]. Oxidative stress occurs if disequilibrium between reactive oxygen species (ROS) production and antioxidative capacity of the cell takes place [19] and it has also been implicated in the etiology of some forms of female infertility [20]. Mitochondria represent the major source of ROS, in which they are produced in a stepwise process with a final reduction of O_2_ to H_2_O during oxidative phosphorylation, in particular at the level of complex I and III [21]. Under physiological conditions, ROS are neutralized by an elaborate defence system consisting of enzymes such as catalase, superoxide dismutase, glutathione peroxidase or reductase and numerous non enzymatic antioxidants such as vitamin C, E, A, pyruvate, glutathione, ubiquinone, taurine and hypotaurine [22]. Thus, any perturbation in mitochondrial or in the activity of scavenger systems can lead to profound implications in ROS production, OS induction, and mitochondrial cytochrome c release, which is an important step for apoptosis [23]. Oocytes, as other aerobic cells, produce ATP and ROS by means of mitochondrial oxidative phosphorylation. Recent studies evidenced that, during functional maturation of the oocyte or in pathological conditions, mitochondria display dynamic tubular networks undergoing fission, fusion, organization in granules and tubules and intracellular movements [24]-[26].

The aim of the present study was to analyze the in vitro effects of DEHP on oocyte maturation, energy and oxidative status in the horse, a large animal model. In order to assess the role of cumulus cells in mediating or counteracting the effects of DEHP, cumulus cell (CC) apoptosis and oxidative status were also assessed.

## Results

The study was conducted in Southern Italy (41^st^ North parallel) during three subsequent breeding seasons. No statistically significant differences were found in all examined parameters as related to cumulus morphology at retrieval, whether compact (Cp) or expanded (Exp), so that data of all cumulus-enclosed oocytes were pooled.

### Experiment 1

In Experiment 1, cytological assessment of CC apoptosis and evaluation of oocyte nuclear maturation rate, mt and ROS distribution pattern, fluorescent intensities and colocalization were performed. DEHP was used at the concentrations of 0.12, 12 and 1200 µM and control oocytes were cultured in the absence of DEHP. Cumulus cell apoptosis was analyzed by Hoechst 33258 staining in order to follow nuclear chromatin fragmentation and condensation as indicators of late-stage chromatin damage. The ovaries of 39 mares were processed. One hundred and twenty five oocytes were recovered (1.6 oocytes/ovary), 67 surrounded by a Cp cumulus and 58 with an Exp cumulus and cultured for IVM in five consecutive trials.

### DEHP induces chromatin fragmentation and condensation in CCs

**Figure 1:**
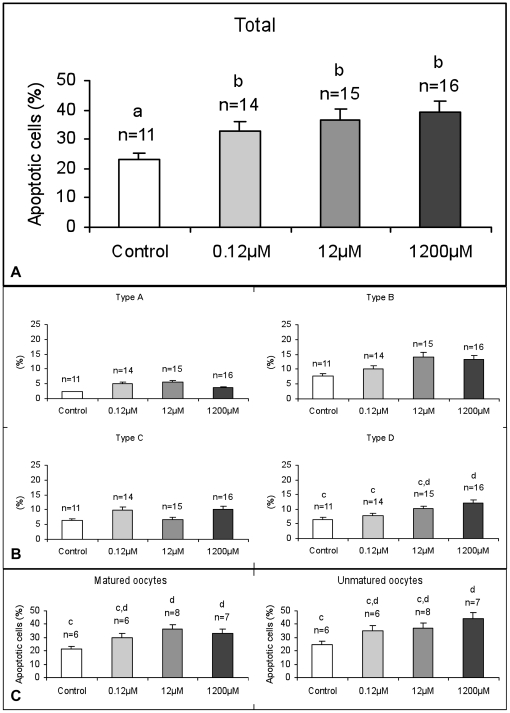
Effects of DEHP on cumulus cell chromatin fragmentation and condensation. At all tested concentration, DEHP increased cumulus cells chromatin fragmentation and condensation in equine COCs (A). In separate evaluation of morphological features of apoptosis, such as marginated chromatin (Type A), single small densely stained nucleus (Type B), multiple densely nuclear fragments (Type C), apoptotic bodies (Type D), a significant increase of apoptotic bodies could be observed after IVM in presence of DEHP (B). Cumulus cell chromatin morphology was affected irrespectively to oocyte maturation stage (C). Numbers of analyzed *cumuli oophori* per group, from which cells analyzed where obtained, are indicated on the top of each histogram. One-way ANOVA: a, b P<0.001; c,d P<0.01.

A clear harmful effect of DEHP on CC nuclear chromatin fragmentation and condensation was visible at all tested concentration (P<0.001; Figure 1A). At higher doses (12 and 1200 µM), DEPH induced a significant increase of late stage apoptotic morphologies (Type D = apoptotic bodies; P<0.01; Figure 1B). Moreover, DEHP was effective at all tested concentrations irrespectively of oocyte nuclear maturation (P<0.01; Figure 1 C).

### DEHP at low doses affects oocyte nuclear maturation

A statistically significant reduction of the maturation rate was observed at 0.12 µM DEHP compared with controls (9/32, 28% vs 16/28, 57%; P<0.05; Table 1). No effects were noticed at the other tested concentrations. In order to evaluate the effects of DEHP on oocyte developmental potential, a set of fluorescent labelling confocal microscopy energy/redox ooplasmic parameters, such as mt distribution pattern, apparent energy status, intracellular ROS localization and levels, were examined in single MII oocytes obtained after IVM in presence of DEHP.

**Table 1:** *In vitro* effects of DEHP on oocyte meiotic maturation.

DEHP concentration (µM)	N. of evaluated oocytes	Nuclear chromatin configuration:			
		Germinal vesicle	Metaphase I to Telophase I	Metaphase II and 1^st^ Polar Body	Abnormal
0	28	0 (0)	7 (25)	16 (57)^ a^	5 (18)
0.12	32	2 (6)	8 (25)	9 (28)^ b^	13 (41)
12	34	3 (9)	3 (9)	18 (53)	10 (29)
1200	31	3 (10)	6 (19)	13 (42)	9 (29)

Chi-square Test: a,b P<0.05.

### DEHP does not affect mitochondrial distribution pattern and ROS intracellular localization in MII oocytes

**Figure 2:**
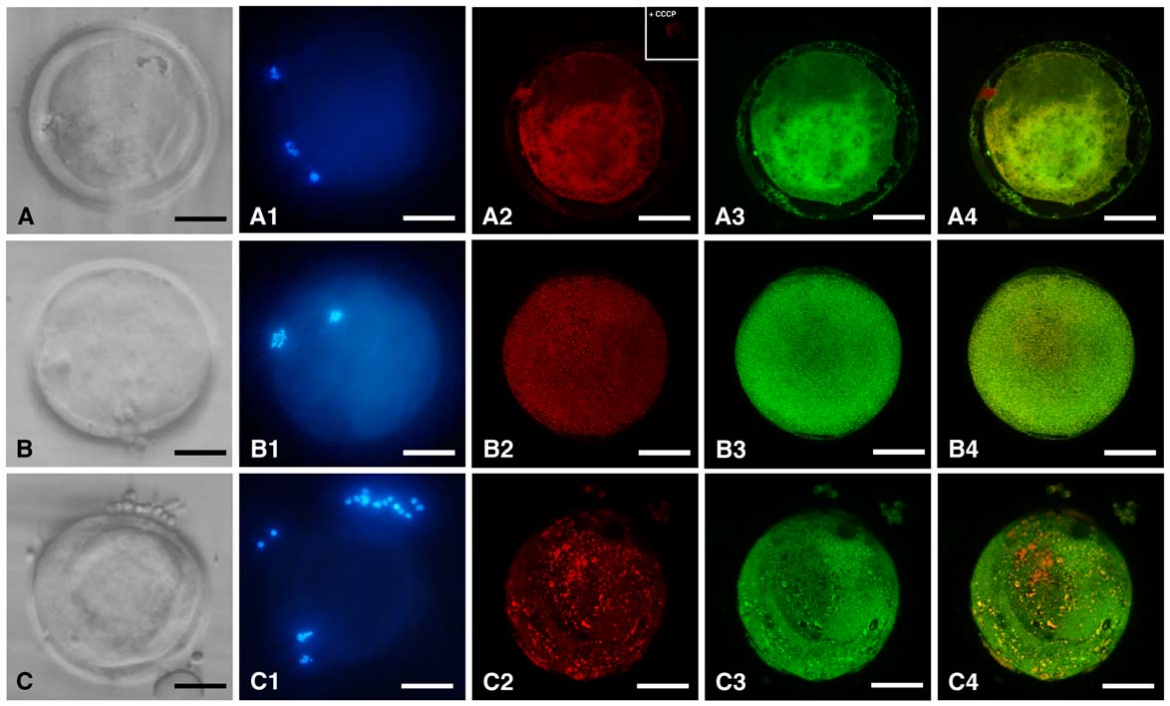
Mitochondrial distribution pattern and ROS localization in equine matured oocytes exposed to DEHP. For each oocyte, corresponding bright-field (A,B,C), UV light (A1,B1,C1) and confocal laser scanning images showing mt distribution pattern (A2,B2,C2), intracellular ROS localization (A3,B3,C3) and mt/ROS merge (A4,B4,C4) are shown. Oocytes are representative of heterogeneous (pericortical/perinuclear; A), homogeneous (B) and abnormal (C) mitochondrial distribution pattern, respectively. Scale bar represents 60 µm.

Mitochondrial distribution pattern did not vary in DEHP-treated oocytes compared with controls (Table 2). Oocytes were found as showing either heterogeneous (pericortical/perinuclear, P/P) or homogeneous or abnormal distribution pattern. Intracellular ROS localization also did not vary upon DEHP exposure. Data concerning ROS localization corresponded to data of mt distribution pattern presented in Table 2. Figure 2 shows matured equine oocytes representative of heterogeneous (A), homogeneous (B) and abnormal (C) mt distribution patterns with corresponding intracellular ROS localization and merge.

**Table 2:** *In vitro* effects of DEHP on oocyte mitochondrial distribution pattern.

DEHP concentration (µM)	N. of oocytes found at the MII stage and evaluated	Mitochondrial distribution pattern		
		Pericortical/Perinuclear	Small aggregates	Abnormal
0	14	7 (50)	5 (36)	2 (14)
0.12	9	6 (67)	3 (33)	0 (0)
12	18	9 (50)	4 (22)	5 (28)
1200	11	7 (64)	2 (18)	2 (18)

Chi-square Test: NS.

### DEHP increased intracellular ROS levels without affecting apparent energy status and mt/ROS colocalization in MII oocytes

**Figure 3:**
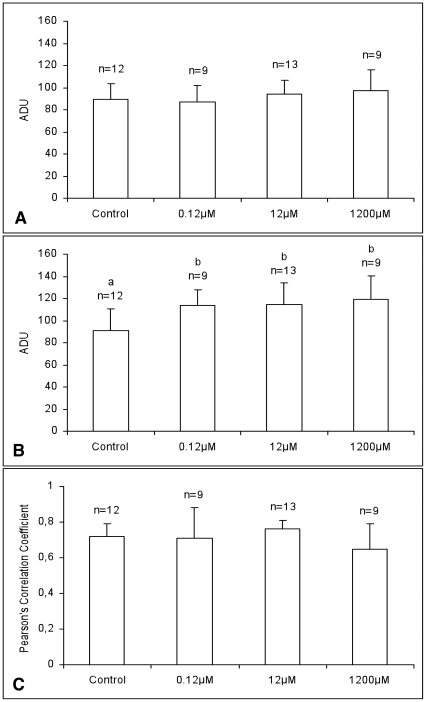
Effects of DEHP on mitochondrial activity, intracellular ROS levels and mt/ROS colocalization in matured oocytes. Dose-response curve of the in vitro effects of DEHP on mitochondrial activity and intracellular ROS levels in single equine metaphase II stage oocytes expressed as Mitotracker Orange CMTM Ros (A) and DCF (B) fluorescence intensities. Oocytes treated with DEHP showed significantly higher ROS levels compared to controls. Values are expressed as arbitrary densitometric units (ADU). Pearson's correlation coefficents of Mitotracker Orange CMTM Ros and DCF fluorescent labelling in oocytes cultured in presence of DEHP (C). Numbers of analyzed oocytes per group are indicated on the top of each histogram. Student's t-Test: a, b P<0.05.

Mitotracker Orange CMTM Ros fluorescence intensity, indicating oocyte apparent energy status, did not vary upon DEHP exposure (Figure 3A). On the contrary, oocytes treated with DEHP at all tested concentrations showed significantly higher values of DCF fluorescence intensity, indicative of intracellular ROS levels, than controls (Figure 3B; P<0.05). At examined concentrations, DEHP did not affect mt/ROS co-localization in MII oocytes (Figure 3C).

### Experiment 2

In Experiment 2, based on the observation that DEHP increased intracellular ROS levels, the effects of DEHP on total ATP content and total SOD activity in single MII oocytes were investigated. DEHP was used at the concentration of 12 µM which was reported to be within the range of real environmental exposure levels [27]. For the evaluation of ATP content, the ovaries of 49 mares were processed. One hundred and seventy six oocytes were recovered (1.8 oocytes/ovary), 84 surrounded by a Cp cumulus and 92 with an Exp cumulus and cultured for IVM, in six trials. For total SOD activity, the ovaries of 23 mares were processed. One hundred and fifteen oocytes were recovered (2.5 oocytes/ovary), 57 surrounded by Cp cumulus and 58 with an Exp cumulus and cultured for IVM in five trials.

### DEHP increases ATP content but does not affect total SOD activity in single MII oocytes

**Figure 4:**
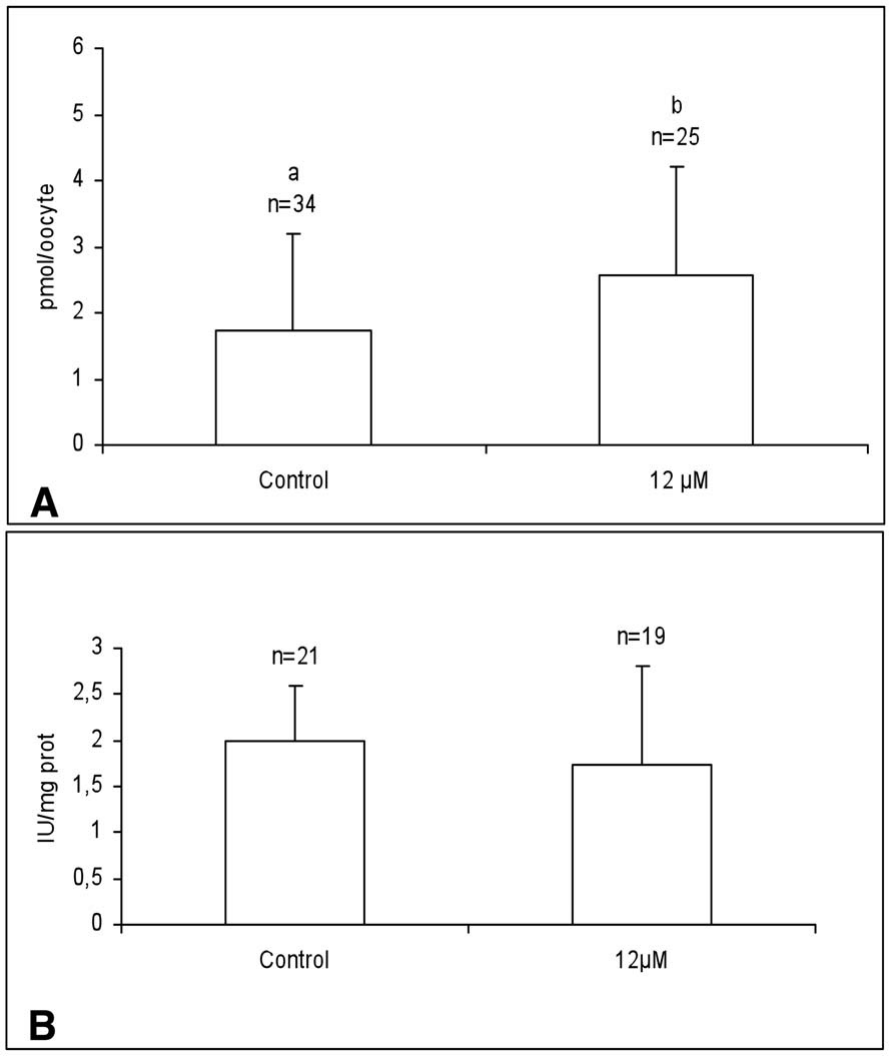
Effects of DEHP on total ATP content and total SOD activity in single equine matured oocytes. ATP content and total SOD activity in single equine MII oocytes cultured in presence of 12 µM DEHP. Control oocytes were cultured in absence of DEHP. Numbers of analyzed oocytes per group are indicated on the top of each histogram. Oocytes treated with 12 µM DEHP showed significantly higher ATP content compared to controls. Values are expressed as pmol/oocyte (A). Total SOD activity did not vary upon 12 µM DEHP exposure in matured equine oocytes compared with controls. Values are expressed as IU/mg protein (mean±sd of 9 evaluations per oocyte; B). Student's t-Test: a, b P<0.05.

The ATP content was significantly higher in oocytes treated with DEHP compared with controls (Figure 4A; P<0.05). The total SOD activity did not vary between DEHP-treated and control oocytes (Figure 4B; NS).

### Experiment 3

Experiment 3 was performed to clarify whether and how the effects of DEHP on CC apoptosis are related to oocyte maturation. To this aim, co-treatments between DEHP and the antioxidant N-Acetyl-Cysteine (NAC) were performed at all tested DEHP concentrations and, after culture, CCs of each COC were simultaneously analyzed for apoptosis and intracellular ROS levels and oocytes were analyzed for nuclear maturation rate and energy/oxidative parameters, as in Experiment 1. In order to evaluate the effect of DEHP on late-stage molecular DNA damage, CC apoptosis was analyzed by Terminal Deoxynucleotidyl Transferase-mediated dUTP Nick-End Labeling (TUNEL). The ovaries of 58 mares were processed. Two hundred and eighty seven oocytes were recovered (2.5 oocytes/ovary), 131 surrounded by a Cp cumulus and 156 with an Exp cumulus and cultured for IVM in three trials.

### NAC reversed DEHP-induced apoptosis in CCs

**Figure 5:**
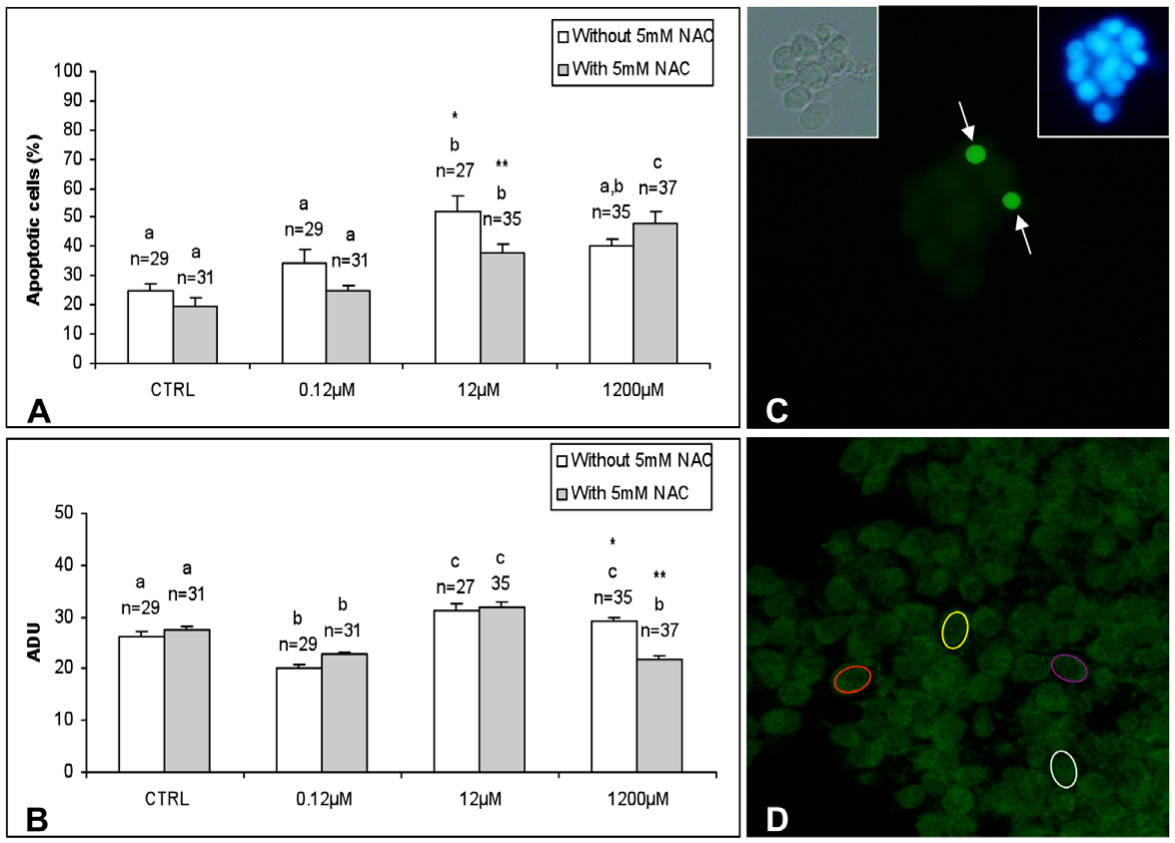
Effects of DEHP and DEHP/NAC co-treatment on cumulus cell apoptosis and intracellular ROS levels. At the concentration of 12 and 1200 µM, DEHP increased cumulus cell apoptosis in equine COCs. NAC reversed DEHP-induced apoptosis observed at 12 µM (A). At the concentrations of 12 and 1200 µM, DEHP increased CC intracellular ROS levels and co-treatment with NAC reduced the DEHP-induced ROS increase observed at 1200 µM (B). Numbers of analyzed *cumuli oophori* per group (from MII+NM oocytes), from which analyzed cells where obtained, are indicated on the top of each histogram. Representative images of equine CCs after IVM in presence of DEHP subjected to TUNEL analysis (C) to determine apoptosis and DCF staining (D) to evaluate intracellular ROS levels. In C, corresponding bright-field and UV light images are also provided. One-way ANOVA: comparisons between used DEHP doses: a,b,c P<0.0001; comparisons between DEHP and DEHP/NAC: _*_,_**_ P<0.0001.

The addition of 12 and 1200 µM DEHP increased CC apoptosis (P<0.0001; Figure 5A,C), thus confirming morphological observations in Experiment 1. At any DEHP tested concentration, the apoptotic index was higher in CCs from oocytes found as non matured (NM) after IVM compared with CCs from MII oocytes (46±22% vs 24±8%) and this difference attained statistical significance at 0.12 µM (P<0.0001; File S1,a). NAC co-treatment was able to counteract DEHP-induced CC apoptosis observed at 12 µM (P<0.0001; Figure 5A) and, at this concentration, the apoptotic index was significantly higher in CCs from NM oocytes compared with CCs from MII oocytes (46±14% vs 29±10%; P<0.0001; File S1,b).

### NAC counteracts intracellular ROS increase in DEHP-exposed CCs

The exposure to 0.12 µM DEHP significantly reduced intracellular ROS levels (P<0.0001) in CCs whereas it increased ROS levels when used at 12 and 1200 µM (P<0.0001; Figure 5B,D). The ROS reduction observed at 0.12 µM DEHP was not related to nuclear maturation of corresponding oocytes (reduced ROS levels were found in CCs from either NM or MII oocytes) whereas the increase observed at 12 and 1200 µM DEHP was significantly higher in CCs from MII oocytes (P<0.0001; File S1,c). Co-treatment with NAC efficiently scavenged excessive ROS caused by DEHP at 1200 µM (P<0.0001) whereas it had no effects at 12 µM (Figure 5B). At this DEHP concentration, ROS levels were significantly higher in CCs from MII oocytes compared with CCs from NM oocytes (P<0.0001; File S1,d).

### Overall assessment of DEHP-induced oocyte damage

Concerning DEHP-induced oocyte damage, overall data (Experiment 1+Experiment 3) are provided. DEHP was shown to inhibit oocyte nuclear maturation only when used at low doses (0.12 µM; 19/61, 31% vs 29/57, 51% for DEHP-treated and control oocytes, respectively; P<0.05) thus confirming the results of experiment 1. At any tested concentration, it had no significant effect on examined microscopy ooplasmic energy/redox parameters (File S2).

### NAC does not improve the maturation rate of DEHP-exposed oocytes

NAC co-treatment tended to reverse the inhibitory effect of DEHP on oocyte maturation (14/31, 45% vs 10/29, 34% for DEHP/NAC- and DEHP-treated oocytes, respectively) but this effect did not attain statistical significance. Interestingly, the addition of NAC “per sé” had a stimulatory effect on oocyte maturation (23/31, 74% vs 13/29, 45% for NAC-treated and control oocytes, respectively; P<0.05).

## Discussion

To our knowledge, this is the first study which analyzes the effects of in vitro acute exposure to DEHP on energy and oxidative parameters of single cumulus-oocyte complexes by using a large animal model. The mare represents a valid model to investigate oocyte physiology due to its particularly large ovarian follicle size, which allows the possibility to relate oocyte meiotic and developmental competence with biochemical and molecular features of corresponding follicle cells as reported in the studies performed by several groups [28]-[35].

As the viability/quality of CCs has been indicated as a crucial factor influencing the outcome of oocyte maturation and subsequent developmental competence, the incidence of apoptotic chromatin modification in CCs after exposure to DEHP has been investigated first. DEHP, at any tested concentration, induced chromatin damage in CCs which was displayed in all characteristics morphological features of apoptosis. On the other hand, the TUNEL assay revealed DEHP-induced DNA fragmentation only at higher tested concentrations (12 and 1200 µM). Thus, chromatin condensation and fragmentation observed after exposure at low DEHP doses (0.12 µM) may not be associated with DNA apoptotic cleavage. In addition, chromatin breakage was observed irrespectively of oocyte maturation, whereas DNA cleavage was higher in CCs from NM oocytes. These observations indicate that these two methods provide complementary information. Our data confirmed previous observations obtained with MEHP. The inhibitory effect of MEHP on maturation was reported to be more severe in denuded oocytes compared to intact COCs, indicating that cumulus cells are primarily affected by MEHP and are able to reduce the harmful effect of MEHP on oocyte maturation [1].

The presence of DEHP at low doses (0.12 µM) in the maturation medium negatively influenced the ability of equine oocytes to reach meiotic maturation at the MII stage. One of the main question involving phthalates, is whether the level of exposure is sufficient to adversely affect female reproductive health. Recently, several studies reported that treatment of rat with active phthalates may result in non-monotonic response curves and low-dose effects [36]-[38]. This is in agreement with results reported in the present study, showing major adverse effects on oocyte maturation at the lowest dose investigated. It is noteworthy to observe that the current no observed adverse effect level (NOAEL) adopted by the European Food Safety Authority for DEHP is 5 mg/Kg/day, based on a multigeneration study using alterations in male reproductive organs as endpoint [39]. However, it is also important to note that the significance of low-dose changes, as observed in the present study, is still largely unknown and, due to difficulties in *in vitro* to *in vivo* extrapolation, cannot be taken as clear evidence for concern about real-life exposure. To address this question, further analysis in animal models are necessary in order to better understand the cellular and molecular mechanisms at the basis of the observed effects. At the moment, few studies have been published regarding the *in vitro* effects of phthalates on oocyte maturation. To our knowledge, only one study reported the *in vitro* effects of DEHP on meiotic maturation [13]. These authors found that DEHP at the concentrations of 0.0001, 0.01, 1 and 100 µM did not affect meiotic stage of porcine oocytes. Another study [1] reported that, in bovine oocytes, MEHP at concentrations between 25 and 100 µM blocked oocytes from reaching the MII stage of maturation. A subsequent study on mouse oocytes [12] confirmed these results, as the proportion of oocytes that progressed to the MII stage was significantly reduced by adding MEHP in a dose-related manner. Mlynarcíková et al. in their study (2009) [13] supposed that the different results obtained in their study may be a consequence of the related but still dissimilar chemical structure of MEHP and DEHP. Our results lead us to hypothesize that different sensitivity to DEHP may be observed in different species.

How phthalates influenced oocyte maturation is not clearly understood. It was reported that MEHP inhibited FSH-stimulated cAMP production in cultured granulosa cells [40] considering that intracellular build up of cAMP is necessary for optimum developmental competence [41], [42]. DEHP/MEHP probably mimic the effects of fatty acids on granulosa cells [6] because they are ligands for fatty acid binding proteins [43], and it has been shown that fatty acids adversely affect in vitro maturation [44], [45]. Phthalates have been involved in PPARs activation, that lead to a decrease in aromatase and estradiol levels [46] and several reports [14] indicate that lower estradiol secretion from granulosa cells is responsible for impaired oocyte maturation. A more recent study [27] indicated that MEHP is a specific inhibitor of estradiol production in human granulosa cells with a post-cAMP site of action. These authors observed the inhibition of estradiol production resulting from a reduction of aromatase activity at the transcript level.

In order to evaluate whether DEHP could affect cytoplasmic maturation, thus developmental potential of exposed oocytes, a set of cytoplasmic energy/redox parameters was investigated in oocytes found at the MII stage after culture in presence of DEHP. In Experiment 1, the addition of DEHP at any used concentration did not affect mt distribution pattern, intracellular ROS localization, apparent energy status and mt/ROS colocalization whereas it increased ROS levels. This observation prompted us to hypothesize that the DEHP-induced ooplasmic ROS levels increase could be responsible for altered oocyte maturation, also in agreement with previous studies reporting that oxidative stress may be an important mechanism underlying the toxic effects of DEHP [18]-[20]. However, 0.12 µM was the only dose that inhibited oocyte maturation while all tested doses increased ooplasmic ROS levels.

The increase of intracellular ROS was supposed to be due to a lack of one or more scavenging enzyme activities or to a loss of function of the mitochondrial respiratory chain proteins, as reported by Bonilla and del Mazo (2010) [47] who showed a deregulation of genes encoding Cu-Zn superoxide dismutase (SOD1) and Nd1 mitochondrial protein upon in vitro exposure to MEHP. In addition, our finding that DEHP did not affect mt/ROS colocalization lead us to hypothesize that ROS produced in excess in DEHP-treated oocytes could be located at mitochondrial level. In order to explore whether increased ROS levels could be due to unefficient scavenging enzyme activity, our subsequent aim (Experiment 2) was to investigate total SOD activity in oocytes found as matured after culture in presence of 12 µM DEHP. To our knowledge, this is the first study reporting total SOD activity in single equine oocytes matured in vitro. The results of our study indicated that SOD activity was not affected in DEHP-exposed oocytes compared with controls. Thus, the intracellular ROS increase observed in DEHP-exposed oocytes could be due to altered efficiency of other antioxidant systems.

Moreover, ATP content analysis showed an increase of ATP levels in MII oocytes obtained after incubation with 12 µM DEHP compared to controls. These data apparently disagree with the results of confocal analysis in which no differences in mt apparent energy status (Mitotracker Orange CMTM Ros fluorescence intensity) were found between treated and control samples. However in the performed test, total ATP levels were measured and it could be possible that the observed ATP increase could be of cytosolic (or glycolitic) origin. To our knowledge the only study about DEHP effects on glycolisis was reported by Martinelli et al. (2006) [48] who found increased pyruvate and lactate content in skeletal muscle after in vivo DEHP administration. The reported increase of pyruvate and lactate, which are responsible for increased cytosolic ATP, could be considered in agreement with our findings.

In order to assess whether the observed inhibitory effect of DEHP on oocyte maturation was related to oxidative stress and apoptosis in the COC, the potential reversibility of DEHP-induced COC damage by the antioxidant NAC was examined. NAC was shown to counteract the damaging effects of the environmental toxicant arsenic trioxide on mitochondrial function in mouse matured oocytes [49]. Apoptotic index and ROS genreation were also reversed by NAC in human ovarian cancer cells treated with WP 631, an anticancer anthracycline analog, and with doxorubicin, the best known first-generation anthracycline [50]. Another recent study [51] reported no differences in nuclear maturation, fertilization and cleavage rate, but higher blastocysts formation rate in presence of NAC compared with controls in porcine oocytes.

It came out that:

- at low doses, DEHP increased CC apoptosis, observed as chromatin condensation/fragmentation but not as DNA cleavage, and reduced CC intracellular ROS levels, thus causing loss of CC viability with consequent inhibitory effect on oocyte nuclear maturation;
- at higher doses, DEHP increased CC apoptosis, at chromatin and DNA level, and increased CC ROS levels but it does not affect oocyte maturation.

In conclusion, our data indicate that DEHP exerts a dose-dependent effect on CC apoptosis and oocyte meiotic maturation which was more deleterious at low doses. This effect is not mediated by cellular stress but rather by loss of CC viability. CC acts as a protection barrier against the harmful effects of DEHP. Energy/redox parameters of MII DEHP-exposed oocytes are consistent with the possibility that they can sustain fertilization and embryo development. These findings means that DEHP-exposed oocytes can be used, with apparent lack of effects on female fertility, but could also represent a potential risk of generating abnormal embryos and this topic needs further investigations. Recognition that the toxic effects of DEHP may originate in disruption of mitochondrial biology, at CCs or ooplasmic level, could represent important findings for the future development of therapeutical procedures to phthalates exposure-derived infertility. The risk of infertility may be reduced by optimizing mitochondrial energy metabolism within the “contaminated oocyte” by new therapeutic skills of nutritional, pharmacological or technological means, such as antioxidants-enriched diets, antioxidants added to culture media for assisted reproductive technologies and homologous mitochondrial ooplasmic transfer.

## Materials and Methods

### Chemicals and culture media

All chemicals were purchased from Sigma–Aldrich (Milano, Italy) unless otherwise indicated.

Medium TCM-199 with Earle's salts, buffered with 4.43 mM HEPES and 33.9 mM sodium bicarbonate and supplemented with 0.1 g/L L-glutamine, 2 mM sodium pyruvate, 2.92 mM calcium-L-lactate pentahydrate (Fluka 21175 Serva Feinbiochem GmbH & Co. Heidelberg, Germany No. 29760) and 50 µg/mL gentamicin was used. After preparation, pH was adjusted to 7.18 and the medium was filtered through 0.22-µm filters (No. 5003-6, Lida Manufacturing Corp., Kenosha WI, USA) and further supplemented with 20% (v/v) Fetal Calf Serum (FCS). Then, gonadotrophins (10 µg/mL ovine FSH and 20 µg/mL ovine LH) and 1 µg/mL 17β Estradiol were added. The medium was filtered again and allowed to equilibrate for 1 h under 5% CO_2_ in air before being used.

### Collection and culture of cumulus-oocyte complexes

Ovaries from mares of unknown reproductive history, obtained at two local abattoirs (Fin.Sud. Import s.r.l. and Maselli Carni Per Te s.r.l.) located at a maximum distance of 30 km (30 min) from the laboratory, were transported and processed for the scraping procedure as previously described [32]. Cumulus-oocyte complexes (COCs) were recovered from medium size follicles (0.5–2.5 cm in diameter), identified in the collected mural granulosa cells by using a dissection microscope and only healthy COCs, classified as having an intact Cp or Exp cumulus investment [30], [32] were selected for culture; degenerating oocytes (having shrunken, dense or fragmented cytoplasm) were recorded and discarded. The time between follicle scraping and beginning of oocyte culture was less than 1 h. Total time between slaughter and culture ranged between 2 and 4 h.

In vitro maturation was performed following the procedure by Dell'Aquila et al. (2003) [32]. Cp and Exp COCs were washed three times in the culture medium and groups of COCs with the same cumulus morphology were placed in 400 µL of medium/well of a four-well dish (Nunc Intermed, Roskilde, Denmark), covered with pre-equilibrated lightweight paraffin oil and cultured for 28–30 h at 38.5°C under 5% CO_2_ in air. DEHP (Sigma Supelco, Cat # 47994) stock solution (1 g/1 ml methanol) was prepared following manufacturers instructions and dilution 1∶20 (v/v) in pure ethanol was performed. The effects of DEHP for the whole duration of IVM culture were tested at final concentrations of 0.12, 12 and 1200 µM. Due to unknown phtalates concentration in equine follicular fluid, the minimum dose has been calculated on the basis of observations on daily dose exposure in humans [52], [53] divided by a factor of 1000. The dose range was then obtained by multiplying the calculated dose by a factor of 100, up to the highest dose which showed reproductive toxicity in previous in vitro studies [1]. Control oocytes were cultured in the absence of DEHP. The N-Acetyl Cysteine was used at the concentration of 5 mM, reported as being effective in counteracting energy and oxidative damage in mouse oocytes [49]. The effects of NAC were analyzed at all DEHP tested concentrations. After IVM, oocytes underwent cumulus and corona cells removal by incubation in TCM 199 with 20% FCS containing 80 IU hyaluronidase/mL and aspiration in and out of finely drawn glass pipettes. Oocyte maturation was initially assessed after denuding by observation under a Nikon SMZ 1500 stereomicroscope (60–110x magnification) evaluating the extrusion of the first polar body (PB) in the perivitelline space and was confirmed by nuclear chromatin evaluation as described below.

### Morphological assessment of cumulus cell apoptosis

Cumulus cells in experiment 1 were fixed overnight at 4°C in 3.8% (v/v) buffered formaldehyde solution (J T Baker; No. 7385) in PBS, then stained with 2.5 mg/ml Hoechst 33258 in 3∶1 (v/v) glycerol/PBS and observed under an E-600 Nikon fluorescent microscope equipped with a 365 nm excitation filter. Morphological criteria for apoptotic cell and bodies described previously were used [54]. Cells were classified in 4 different categories: A, B, C and D. Type A: cells with nuclei containing marginated chromatin; type B: cells with a single small nucleus with densely stained chromatin; type C: cells containing multiple nuclear fragments and type D: membrane-bound structures containing variable amount of chromatin and/or cytoplasm (apoptotic bodies). Evaluation of apoptotic cells and apoptotic bodies was performed on a mean of 10 to 15 COCs per culture condition. For each COC, two fields with 50 cumulus cells each were countered.

### Terminal Deoxynucleotidyl Transferase-mediated dUTP Nick-End Labeling (TUNEL)

To assess the rate of apoptotic cells, CCs were separated in groups according to the treatments and oocyte nuclear maturation. Briefly, CCs were fixed in 2% PBS-buffered paraformaldehyde over night at 4°C. An in situ cell death detection kit (Roche Molecular Biochemicals, code: 11684795910; Mannheim, Germany) was used for labeling apoptotic cells. CCs were washed three times in PBS and then permeabilized with 0.5% Triton X-100, 0.1% sodium citrate in PBS for 10 min. CCs were washed twice with PBS before labeling. The TUNEL reagent was prepared immediately before use and kept on ice. CCs were placed in 50 µl drops of TUNEL reagent and incubated in the dark for 1 h at 37°C in a humidified chamber. After incubation, CCs were washed three times with PBS. Total cell nuclei were stained with 10 µg/ml Hoechst 33258, 2.3% Na-citrate in 3∶1 (v/v) glycerol/PBS, mounted on microscope slides, covered with cover-up micro slides, sailed with nail polish and kept at 4°C in the dark until observation. CCs were observed under an E-600 Nikon fluorescent microscope equipped with a 365 nm excitation filter. Positive and negative controls were performed following the manufacturers instructions. Apoptosis was determined as the percentage of labeled cells to the total cell number. For each culture condition, a minimum of 1000 randomly chosen cells was examined.

### Mitochondrial and ROS staining

Oocytes were washed three times in PBS with 3% bovine serum albumin (BSA) and incubated for 30 min in the same medium containing 280 nM MitoTracker Orange CMTM Ros (Molecular Probes M-7510, Oregon, USA) at 38.5°C under 5% CO_2_. The cell-permeant probe contain a thiol-reactive chloromethyl moiety. Once the MitoTracker probe accumulates in the mitochondria, it can react with accessible thiol groups on peptides and proteins to form an aldehyde-fixable conjugate. This cell-permeant probe is readily sequestered only by actively respiring organelles depending on their oxidative activity [55], [56]. After incubation with MitoTracker Orange CMTM Ros, oocytes were washed three times in PBS with 0.3% BSA and incubated for 15 min in the same media containing 10 µM 2′,7′-dichlorodihydrofluorescein diacetate (DCDHF DA). The non-ionized DCDHF DA is membrane permeant and therefore is able to diffuse readily into cells. Once within the cell, the acetate groups are hydrolysed by intracellular esterase activity forming 2′,7′-dichlorodihydrofluorescein (DCDHF) which is polar and thus trapped within the cell. DCHF fluoresces when it is oxidized by H_2_O_2_ or lipid peroxides to yield 2′,7′-dichlorofluorescein (DCF). The level of DCF produced within the cells is linearly related to that of peroxides present and thus its fluorescent emission provides a measure of the peroxide levels [57]. After incubation, oocytes were washed three times in prewarmed PBS without BSA and fixed overnight at 4°C with 2% paraformaldehyde solution in PBS. The organelle-specificity of the probe was assessed, as reported by Valentini et al., (2010) [58], in control samples which were imaged after incubation in MitoTracker Orange and further incubation for 5 min in the presence of 5 µM of the mt membrane potential (Delta Psi)-collapsing uncoupler carbonyl cianide 3-chloro phenylhydrazone (CCCP; Molecular Probes), which inhibits mt respiratory activity thus reducing fluorescence intensity. Cumulus cells were stained with DCDHF DA in order to evaluate intracellular ROS levels. Particular attention was paid to avoid sample exposure to the light during staining and fixing procedures in order to reduce photobleaching.

### Assessment of oocyte nuclear maturation

To evaluate nuclear chromatin, oocytes were stained with 2.5 mg/ml Hoechst 33258 in 3∶1 (v/v) glycerol/PBS, mounted on microscope slides, covered with cover-up micro slides, sailed with nail polish and kept at 4°C in the dark until observation. Nuclear chromatin status was observed under a Nikon Eclipse 600 fluorescent microscope equipped with B2A (346 nm excitation/460 nm emission) filter and classified as follows: GV including those oocytes with fluorescent nucleus and those with a condensed chromatin, metaphase to telophase I (MI to TI) and complete maturation at metaphase II with the first polar body extruded (MII+PB) [59]. Oocytes with irregular chromatin distribution or non detected chromatin were considered as abnormal.

### Assessment of mitochondrial distribution pattern and intracellular ROS localization in matured oocytes

For mt distribution pattern evaluation, oocytes were selected among those having regular ooplasmic size and texture (no vacuoles). Oocytes were observed at 600 x magnification in oil immersion with Nikon C1/TE2000-U laser scanning confocal microscope. A helium/neon laser ray at 543 nm and the G-2 A filter (551 nm exposure/576 nm emission) was used to point out the MitoTracker Orange CMTM Ros. An argon ions laser ray at 488 nm and the B-2 A filter (495 nm exposure/519 nm emission) was used to point out the DCF. Scanning was conducted with 25 optical series from the top to the bottom of the oocyte with a step size of 0.45 µM to allow three-dimensional distribution analysis. General criteria for mt pattern definition were adopted on the basis of previous studies in equine oocytes as well as in other species [59]. Thus, homogeneous/even distribution of small mt granules throughout the cytoplasm was considered as an indication of immature cytoplasmic condition. Heterogeneous/uneven distribution of small and/or large mt granules indicated metabolically active ooplasm. In particular, accumulation of active mitochondria in the peripheral cytoplasm (pericortical mt pattern) and/or around the nucleus (perinuclear and pericortical/perinuclear mt pattern, P/P) was considered as characteristic of full cytoplasmic maturation. Oocytes showing irregular distribution of large mt clusters unrelated to the specific cell compartments were classified as abnormal. To our knowledge, few studies are reported to date on intracellular ROS localization in mammalian oocytes. A recent study performed in mouse oocytes reported that regions producing high levels of ROS colocalized with the active mitochondria in the majority of in vivo matured ovulated oocytes [60]. All oocytes found at the MII stage were analyzed.

### Quantification of Mitotracker Orange CMTM Ros and DCF fluorescence intensity in matured oocytes

Measurements of fluorescence intensities were performed in oocytes having either heterogeneous (pericortical/perinuclear) or homogeneous (small granules) mt distribution pattern. Oocytes showing abnormal mt distribution pattern were excluded from this analysis. In each individual oocyte, the fluorescence intensity was measured at the equatorial plane (plane no. 13), with the aid of the EZ-C1 Gold Version 3.70 software platform for Nikon C1 confocal microscope. A circle of an area (arbitrary value = 100 in diameter) was drawn in order to measure only the cytoplasmic area. Fluorescence intensity encountered within the programmed scan area was recorded and plotted against the conventional pixel unit scale (0–255). Parameters related to fluorescence intensity were maintained at constant values for all evaluations. In detail, images were taken under fixed scanning conditions with respect to laser energy, signal detection (gain) and pinhole size. Based on the observation that co-localization of actively respiring mitochondria and ROS is considered as indication of healthy cell [60], [61], Pearson's correlation coefficient, which describes the correlation of the intensity distribution between channels [62], was used to quantify mt/ROS colocalization as related to DEHP treatment.

### Quantification of DCF fluorescence intensity in CCs

Measurements of DCF fluorescence intensity were performed in CCs isolated from matured and non matured COCs as assessed by PB extrusion evaluation. For each COC, fluorescence intensity was measured on a minimum number of 50 individual randomly chosen cells, with the aid of the EZ-C1 software. A circle was drawn in order to measure the cytoplasmic area of 10 cells and measures were repeated for five fields. Fluorescence intensity encountered within the programmed scan area was recorded and plotted against the conventional pixel unit scale (0–255). Parameters related to fluorescence intensity were maintained at constant values for all evaluations as reported for oocyte evaluations.

### Measurement of the ATP content of single matured oocytes

The ATP content of denuded matured oocytes, cultured with 0 and 12 µM DEHP, was measured using a commercial assay (based on the luciferin-luciferase reaction, ATP lite Perkin Elmer, Monza, Italy). Briefly, after IVM samples were rinsed in TCM 199 supplemented with 20% FCS and then transferred individually in 100 µl of the same medium into plastic tubes. Then, 50 µl of mammalian cell lysis solution was added, and the tubes were kept in the darkness for 5 minutes at room temperature in an orbital shaker. Subsequently, 50 µl of substrate solution was added and after 5 minutes the measurement of the luminescence was performed. The ATP content of the samples was measured using a luminometer (Victor™ X, Perkin Elmer) with high sensitivity (0.01 pmol). A seven point standard curve (0-6 pmol/tube) was routinely included in each assay. The ATP content was determined from the formula for the standard curve (linear regression) [25].

### Measurement of the total superoxide dismutase (SOD) activity in single matured oocytes

The SOD activity was determined on MII oocytes, cultured in presence of 12 µM DEHP. Control oocytes were cultured in absence of DEHP. Single oocytes were previously treated with SB buffer (TRIS/HCl 60 mM pH 6.8, Glycerol 40%) and solubilized for one hour at 4°C in the presence of 1.0% Triton X-100. The protein concentration was assessed by the method of Bradford [63]. Each test was performed on 7 µg of proteins of a single solubilized oocytes. The superoxide dismutase activity was determined with the *Fluka analytical* assay kit using a spectrophotometer Victor™ X, Perkin Elmer at λ = 440 nm. Total SOD activity (SOD, EC 1.15.1.1) was assayed by its ability to inhibit the reduction of a novel tetrazolium salt, WST-1 [2-(4-Iodophenyl)-3-(4-nitrophenyl)-5-(2,4-disulfophenyl)-2H-tetrazolium,monosodium salt] by superoxide anions generated with the xanthine/xanthine oxidase method [64], [65]. One unit of SOD activity was defined as the amount of the enzyme causing half maximum inhibition of WST-1 reduction. It was measured with high sensitivity (0.01 pmol) and expressed as U/mg proteins. A nine point standard curve was routinely included in each assay.

### Statistical analysis

The apoptotic index and intracellular ROS levels in CCs surrounding MII and immature oocytes were analyzed by using GraphPad Prism software (GraphPad Software 5.03, San Diego CA). The percent number of apoptotic cells per cumulus was calculated using oocytes from at least 5 different trials for morphological analysis and 3 trials for TUNEL test. Differences between the means were evaluated by one-way ANOVA, with statistical significance assigned at P 0.05. When a significant *P* value was obtained with ANOVA, the Bonferroni test was used in the post hoc analysis. Oocyte nuclear maturation rates and the rates of oocytes showing the different mt distribution patterns and ROS intracellular localization were compared between treated and control groups by χ^2^-analysis with the Yates correction for continuity. Fisher's exact test was used when a value of < 5 was expected in any cell. For confocal quantitative analysis of mt and ROS fluorescence intensity, the least-square means of the dependent variables (mt and ROS fluorescence intensity) were calculated in examined samples and the statistical significance of the least-square means between control and treated groups was calculated by the Student's t-test. Mean values of Pearson's correlation coefficient, ATP content and SOD activity were compared between treated and control groups by the Student's t-test. Differences with P<0.05 were considered statistically significant.

## Supporting Information

File S1 **Apoptotic index of CCs isolated from matured (MII) and non matured (NM) oocytes after IVM in presence of DEHP (a) and DEHP+NAC (b), as assessed by TUNEL test.** Intracellular ROS levels of CCs isolated from MII and NM oocytes after IVM in presence of DEHP (c) and DEHP+NAC (d), as assessed by DCF fluorescence intensity.
(XLS)

File S2 **Overall assessment (Experiment 1+Experiment 3) of DEHP effects on oocyte apparent energy status, intracellular ROS levels and mt/ROS colocalization as assessed at ooplasmic level in individual MII oocytes.** Number of examined oocytes are reported at the top of each histogram.
(XLS)
