# Correction: In Vitro Acute Exposure to DEHP Affects Oocyte Meiotic Maturation, Energy and Oxidative Stress Parameters in a Large Animal Model

**DOI:** 10.1371/annotation/4eeebe50-1b31-422c-94a0-effcf9eda85f

**Published:** 2011-11-18

**Authors:** Barbara Ambruosi, Manuel Filioli Uranio, Anna Maria Sardanelli, Paola Pocar, Nicola Antonio Martino, Maria Stefania Paternoster, Francesca Amati, Maria Elena Dell'Aquila

The correct Financial Disclosure is: "This work was supported by the Academic Center of Excellence “Comparative Genomics: Genes Involved in Physiopathological Processes in the Biomedical and Agricultural Fields” University of Bari Aldo Moro, Italy; and by the projects: COFIN PRIN MIUR 2007 University of Bari Aldo Moro, Italy cod. 2007S75KSE_003 (Resp. Sci. M.E. Dell’Aquila), COFIN PRIN MIUR 2008 University of Bari Aldo Moro, Italy cod. 2008YTYNKE_001 (Resp. Sci. M.E. Dell’Aquila) and Finanziamenti d’Ateneo 2010 University of Bari Aldo Moro, Italy cod. ORBA10YTAK (Resp. Sci. M.E. Dell’Aquila). The funders had no role in study design, data collection and analysis, decision to publish, or preparation of the manuscript."

There was an error in affiliation 2 for authors Anna Maria Sardanelli, Maria Stefania Paternoster, and Francesca Amati. The correct affiliation 2 is: "Department of Basic Medical Sciences, University of Bari Aldo Moro, Bari, Italy". 

